# Disaster management and primary health care: implications for medical education

**DOI:** 10.5116/ijme.5a07.1e1b

**Published:** 2017-11-30

**Authors:** Javeria Majeed Swathi, Pedro Arcos González, Rafael Castro Delgado

**Affiliations:** 1 Unit for Research in Emergency and Disaster, Faculty of Medicine, University of Oviedo, Spain

**Keywords:** Disaster management, primary health care, implications, medical education, Spain

## Introduction

Natural and manmade disasters often result in the breakdown of social system and services with pronounced effects on human development and economy. They also cause ill-health and deaths either directly or through the disruption of health systems, leaving the affected communities without access to healthcare in times of emergency. Empirical evidence shows that these negative effects are disproportionally concentrated in the developing countries which accounts for 68.2% of globally reported disaster mortalities in 2012.^1^ The geophysical risks, urbanization, population growth and climate changes further increase the vulnerability to natural disasters, particularly, in developing countries.

The increased scale, frequency, and impact of natural and manmade disasters underpin the need for adaptation of context-specific, multi-sectoral and multidisciplinary disaster management interventions and plan. The plan shall encompass the coordination and integration of activities necessary to build, sustain and improve the capability to prepare for, protect against, respond to, and recover from any emergency. Moreover, it is essential to maintain a surge capacity at a local and national level to respond immediately when a health emergency or disaster struck. To this end, high-income countries have established efficient and effective emergency medical care systems, namely, Rescue 112. This system has played a crucial role in responding immediately and successfully managing medical emergencies such as injuries, trauma and other life-threatening conditions. However, establishing such a robust emergency medical care system in low-income countries is not possible due to substantial financial, human and material resources required to maintain and operate such services.^2^ Instead the primary health care exits in low-income countries that provides an opportunity to integrate and mainstream disaster response services. The integration of the disaster management within the primary health care can be proved instrumental in the provision of optimal and low-cost emergency medical assistance by utilizing the existing primary health care network (physical infrastructure and human/financial capital). Additionally, the integration will pave a way in preparing households, communities and health systems in managing disaster related risks and hazards.^3^ Despite the need for adapting integrated approaches, primary healthcare and emergency medical assistance within the broader domain of disaster management have been portrayed and perceived as two separate entities with arguments in favour for and against each.^4^ These arguments revolve around the conceptual definitions whereas primary health care and emergency medical assistance are considered as developmental and emergency response intervention respectively.

Considering the need for access to and availability of emergency medical assistance in the resource-poor settings, this paper discusses the implications for medical education with regard to: a) integration of disaster management in primary health care, and b) minimum disaster management competencies and skills required for physician/general practitioners working in the primary health care facilities.

### Disaster management and primary health care

The learnings from the implementation of the emergency response highlight the need for sustaining all the essential components of primary health care during disasters and adapting a holistic approach by mainstreaming and integrating emergency medical assistance and disaster response into primary health care policies, strategies, and services.^4^^-^^7^ For instance, the humanitarian organization (International Federation of Red Cross and Non-Governmental Organizations) and United Nations in partnership with local government have implemented an array of emergency response programme across the world. At large, this medical emergency response programme has been designed and implemented using the primary health care system and sustaining all its components.^5^ The evidence from implementation also shows that the provision of such an integrated healthcare services from primary health care facilities during disasters has drastically reduced the associated mortalities and morbidities.^3^ Additionally, those organizations who have implemented standalone emergency response services (such as mental health, psychosocial support, immunization, management of exacerbated chronic hypertension and trauma care etc. ) during a disaster correspond to one out of the eight listed components of the primary health care.^5^^-^^9^ The lesson learned from the implementation of standalone emergency response substantiate the need for access to and availability of integrated primary healthcare services to cater the health needs of the affected population optimally. For example, more than 90% of the patients were enrolled and examined for non-surgical primary care in the trauma unit established by army medical core for provision of specialized surgical care, in the advent of earthquake in Pakistan.^10^

### Skills and competencies for disaster management

Further to the need for adaptation of integrated primary health care services, lack of context and healthcare discipline specific disaster management competencies and skills makes another major barrier to the provision of optimal healthcare during disasters. Despite the increased scale and frequency of disasters, limited attention has been made to identify and list the disaster management related core competencies and skills for the health professional. The situation is further precarious with regard to medical/health profession students as nothing has been done in a systematic manner that enables them to meet disaster management related occupational competencies and standards upon their graduation from health professional schools and universities. Primarily, the available limited opportunities such as web-based training programs and conferences target the existing health professional already in practice instead of the health profession students.

More recently, a group of experts from four academic institutions (College of Physicians and Surgeons, the School of Oral and Dental Surgery, the School of Nursing, and the Mailman School of Public Health) worked jointly to develop a list of core competencies in the domain of disaster management. These competencies cover aspects of emergency preparedness, disaster response and disaster risk reduction to better equip first-level-healthcare provider (doctor, midwives, nurses, pharmacist, etc.), with the varying level of proficiency for each cadre. The listed competencies can be broadly categorized into three domains as a) Disaster/Emergency Preparedness, Early Warning and Response system, b) Patient care and Mass Casualty Management, and c) Resource (human and material) Management and Eviction. This serves the foundation to define emergency and disaster preparedness competencies and skills for health profession students.^7^

## Conclusions

Primary health care system exists across all the countries in varying forms and strengthening it further within the sphere of disaster management, particularly in low-income countries, can play a crucial role in building the local capacity, responding immediately when a disaster or public health emergency struck and making the communities more resilient to disasters. However, there is yet a need to integrate and mainstream optimally emergency medical assistance within the primary health care system.

Further to the adaptation of holistic and integrated approaches, it is of paramount importance to develop capacities of first level healthcare providers, managers and health profession students on disaster management related competencies and skills. This signifies the need for medical educators to adapt a tailored contextual strategy for transferring the competencies, under the above listed three domains, to the healthcare providers during their pre-service and in-service training. The defining characteristics of the strategy include but not limited to: mapping these competencies against the existing curriculum in the health professional schools and updating it, developing tailored modules for teacher and faculty members on different aspects of disaster management, arranging training courses for the faculty members to better equip them in transferring these newly acquired skills to the health profession students, developing web-based training programmes, and offering relevant continued medical education courses. Imparting pre-and-in-service training on the standardized competencies will adequately equip and prepare the first level healthcare providers in managing disaster affected patients and address associated ramifications/consequences of any catastrophic event. These competencies can further be institutionalized into the health system and built into the job descriptions/role of the healthcare providers and managers.

### Conflict of Interest

The authors declare that they have no conflict of interest.
